# 5-Chloro-2-(4-chloro­phen­yl)-7-methyl-3-methyl­sulfinyl-1-benzofuran

**DOI:** 10.1107/S1600536810006823

**Published:** 2010-02-27

**Authors:** Hong Dae Choi, Pil Ja Seo, Byeng Wha Son, Uk Lee

**Affiliations:** a Department of Chemistry, Dongeui University, San 24 Kaya-dong Busanjin-gu, Busan 614-714, Republic of Korea; b Department of Chemistry, Pukyong National University, 599-1 Daeyeon 3-dong, Nam-gu, Busan 608-737, Republic of Korea

## Abstract

In the title compound, C_16_H_12_Cl_2_O_2_S, the O atom and the methyl group of the methyl­sulfinyl substituent lie on opposite sides of the plane through the benzofuran fragment. The 4-chloro­phenyl ring is rotated out of the benzofuran plane, as indicated by the dihedral angle of 15.91 (4)°. In the crystal, mol­ecules are linked into chains along the *b* axis by weak inter­molecular C—H⋯O hydrogen bonds.

## Related literature

For the crystal structures of similar 2-(4-fluoro­phen­yl)-5-halo-3-methyl­sulfinyl-1-benzofuran derivatives, see: Choi *et al.* (2009 ▶, 2010*a* ▶,*b* ▶). For the biological activity of benzofuran compounds, see: Aslam *et al.* (2006 ▶); Galal *et al.* (2009 ▶); Khan *et al.* (2005 ▶). For natural products with benzofuran rings, see: Akgul & Anil (2003 ▶); Soekamto *et al.* (2003 ▶).
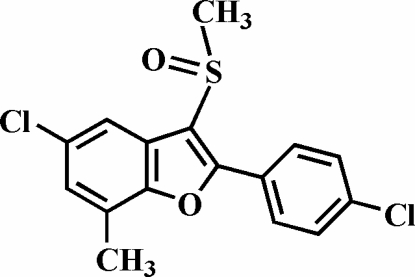


## Experimental

### 

#### Crystal data

C_16_H_12_Cl_2_O_2_S
*M* *_r_* = 339.22
Triclinic, 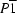

*a* = 7.538 (2) Å
*b* = 9.734 (2) Å
*c* = 11.277 (3) Å
α = 72.525 (2)°
β = 88.846 (3)°
γ = 70.058 (2)°
*V* = 738.8 (3) Å^3^
*Z* = 2
Mo *K*α radiation
μ = 0.58 mm^−1^
*T* = 173 K
0.50 × 0.50 × 0.45 mm

#### Data collection

Bruker SMART APEXII CCD diffractometer
Absorption correction: multi-scan (*SADABS*; Bruker, 2009 ▶) *T*_min_ = 0.760, *T*_max_ = 0.780
6331 measured reflections
3170 independent reflections
2879 reflections with *I* > 2σ(*I*)
*R*_int_ = 0.017

#### Refinement

*R*[*F* ^2^ > 2σ(*F* ^2^)] = 0.031
*wR*(*F* ^2^) = 0.089
*S* = 1.03
3170 reflections
192 parameters
H-atom parameters constrained
Δρ_max_ = 0.67 e Å^−3^
Δρ_min_ = −0.33 e Å^−3^

### 

Data collection: *APEX2* (Bruker, 2009 ▶); cell refinement: *SAINT* (Bruker, 2009 ▶); data reduction: *SAINT*; program(s) used to solve structure: *SHELXS97* (Sheldrick, 2008 ▶); program(s) used to refine structure: *SHELXL97* (Sheldrick, 2008 ▶); molecular graphics: *ORTEP-3* (Farrugia, 1997 ▶) and *DIAMOND* (Brandenburg, 1998 ▶); software used to prepare material for publication: *SHELXL97*.

## Supplementary Material

Crystal structure: contains datablocks global, I. DOI: 10.1107/S1600536810006823/hg2648sup1.cif

Structure factors: contains datablocks I. DOI: 10.1107/S1600536810006823/hg2648Isup2.hkl

Additional supplementary materials:  crystallographic information; 3D view; checkCIF report

## Figures and Tables

**Table 1:** Hydrogen-bond geometry (Å, °)

*D*—H⋯*A*	*D*—H	H⋯*A*	*D*⋯*A*	*D*—H⋯*A*
C15—H15*A*⋯O2^i^	0.96	2.52	3.216 (2)	130

Symmetry code: (i) 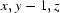.

**Fig. 1.**
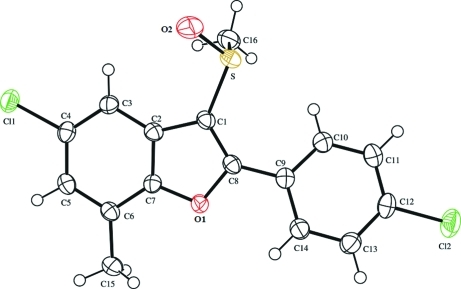
The molecular structure of the title compound with the atom numbering scheme. Displacement ellipsoids are drawn at the 50 % probability level. H atoms are presented as a small spheres of arbitrary radius.

**Fig. 2.**
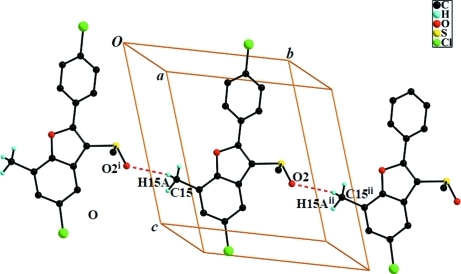
C—H···O interactions (dotted lines) in the crystal structure of the title compound. [Symmetry codes: i) x, y - 1, z; ii) x, y + 1, z.]

## supplementary crystallographic information

### Comment

Compounds containing benzofuran skeleton display significant biological activities such as antifungal (Aslam *et al.*, 2006), antitumor and antiviral (Galal *et al.*, 2009), antimicrobial (Khan *et al.*, 2005) properties. These compounds are widely occurring in nature (Akgul & Anil, 2003; Soekamto *et al.*, 2003). As a part of our ongoing studies of the effect of side chain substituents on the solid state structures of 2-(4-fluorophenyl)-5-halo-3-methylsulfinyl-1-benzofuran analogues (Choi *et al.*, 2009, 2010*a,b*), we report the crystal structure of the title compound (Fig. 1).

The benzofuran unit is essentially planar, with a mean deviation of 0.013 (1) Å from the least-squares plane defined by the nine constituent atoms. The dihedral angle formed by the plane of the benzofuran and the 4-chlorophenyl ring is 15.91 (4)°. The crystal packing (Fig. 2) is stabilized by a weak intermolecular C—H···O hydrogen bond between the methyl H atom and the oxygen of the S═O unit, with a C15—H15A···O2^i^ (Table 1).

### Experimental

77% 3-Chloroperoxybenzoic acid (247 mg, 1.1 mmol) was added in small portions to a stirred solution of 5-chloro-2-(4-chlorophenyl)-7-methyl-3-methylsulfanyl-1-benzofuran (323 mg, 1.0 mmol) in dichloromethane (30 mL) at 273 K. After being stirred at room temperature for 4h, the mixture was washed with saturated sodium bicarbonate solution and the organic layer was separated, dried over magnesium sulfate, filtered and concentrated at reduced pressure. The residue was purified by column chromatography (hexane–ethyl acetate, 1:1 v/v) to afford the title compound as a colorless solid [yield 79%, m.p. 456–457 K; R_f_ = 0.62 (hexane–ethyl acetate, 1:1 v/v)]. Single crystals suitable for X-ray diffraction were prepared by slow evaporation of a solution of the title compound in tetrahydrofuran at room temperature.

### Refinement

All H atoms were positioned geometrically and refined using a riding model, with C—H = 0.93 Å for aryl and 0.96 Å for methyl H atoms. *U*_iso_(H)  = 1.2*U*_eq_(C) for aryl and 1.5*U*_eq_(C) for methyl H atoms.

### Crystal data

**Table:**

C_16_H_12_Cl_2_O_2_S	*Z* = 2
*M**_r_* = 339.22	*F*(000) = 348
Triclinic, *P*1	*D*_x_ = 1.525 Mg m^−^^3^
Hall symbol: -P 1	Mo *K*α radiation, λ = 0.71073 Å
*a* = 7.538 (2) Å	Cell parameters from 5112 reflections
*b* = 9.734 (2) Å	θ = 2.3–27.5°
*c* = 11.277 (3) Å	µ = 0.58 mm^−^^1^
α = 72.525 (2)°	*T* = 173 K
β = 88.846 (3)°	Block, colourless
γ = 70.058 (2)°	0.50 × 0.50 × 0.45 mm
*V* = 738.8 (3) Å^3^	

### Data collection

**Table:**

Bruker SMART APEXII CCD diffractometer	3170 independent reflections
Radiation source: Rotating Anode	2879 reflections with *I* > 2σ(*I*)
Bruker HELIOS graded multilayer optics	*R*_int_ = 0.017
Detector resolution: 10.0 pixels mm^-1^	θ_max_ = 27.0°, θ_min_ = 2.3°
φ and ω scans	*h* = −9→9
Absorption correction: multi-scan (*SADABS*; Bruker, 2009)	*k* = −12→12
*T*_min_ = 0.760, *T*_max_ = 0.780	*l* = −14→14
6331 measured reflections	

### Refinement

**Table:**

Refinement on *F*^2^	Primary atom site location: structure-invariant direct methods
Least-squares matrix: full	Secondary atom site location: difference Fourier map
*R*[*F*^2^ > 2σ(*F*^2^)] = 0.031	Hydrogen site location: difference Fourier map
*wR*(*F*^2^) = 0.089	H-atom parameters constrained
*S* = 1.03	*w* = 1/[σ^2^(*F*_o_^2^) + (0.0425*P*)^2^ + 0.4419*P*] where *P* = (*F*_o_^2^ + 2*F*_c_^2^)/3
3170 reflections	(Δ/σ)_max_ < 0.001
192 parameters	Δρ_max_ = 0.67 e Å^−^^3^
0 restraints	Δρ_min_ = −0.33 e Å^−^^3^

### Special details

**Table:**

Geometry. All esds (except the esd in the dihedral angle between two l.s. planes) are estimated using the full covariance matrix. The cell esds are taken into account individually in the estimation of esds in distances, angles and torsion angles; correlations between esds in cell parameters are only used when they are defined by crystal symmetry. An approximate (isotropic) treatment of cell esds is used for estimating esds involving l.s. planes.
Refinement. Refinement of F^2^ against ALL reflections. The weighted R-factor wR and goodness of fit S are based on F^2^, conventional R-factors R are based on F, with F set to zero for negative F^2^. The threshold expression of F^2^ > 2sigma(F^2^) is used only for calculating R-factors(gt) etc. and is not relevant to the choice of reflections for refinement. R-factors based on F^2^ are statistically about twice as large as those based on F, and R- factors based on ALL data will be even larger.

### Fractional atomic coordinates and isotropic or equivalent isotropic displacement parameters (Å^2^)

**Table:**

	*x*	*y*	*z*	*U*_iso_*/*U*_eq_	
Cl1	0.54735 (6)	0.25715 (5)	1.03989 (4)	0.03540 (12)	
Cl2	−0.06257 (7)	0.78823 (6)	−0.05751 (4)	0.04700 (14)	
S	0.19164 (6)	0.77859 (5)	0.56104 (4)	0.03206 (12)	
O2	0.3263 (2)	0.78670 (16)	0.65093 (15)	0.0479 (4)	
O1	0.31330 (15)	0.36506 (12)	0.52197 (10)	0.0257 (2)	
C1	0.2500 (2)	0.58258 (17)	0.57346 (15)	0.0256 (3)	
C2	0.3368 (2)	0.45451 (18)	0.68431 (15)	0.0251 (3)	
C3	0.3874 (2)	0.43701 (19)	0.80827 (15)	0.0276 (3)	
H3	0.3628	0.5210	0.8373	0.033*	
C4	0.4757 (2)	0.28803 (19)	0.88482 (15)	0.0276 (3)	
C5	0.5149 (2)	0.15889 (18)	0.84399 (15)	0.0277 (3)	
H5	0.5761	0.0613	0.8998	0.033*	
C6	0.4640 (2)	0.17381 (18)	0.72181 (15)	0.0259 (3)	
C7	0.3749 (2)	0.32444 (18)	0.64609 (14)	0.0240 (3)	
C8	0.2391 (2)	0.52362 (17)	0.47881 (15)	0.0251 (3)	
C9	0.1655 (2)	0.58997 (18)	0.34785 (15)	0.0260 (3)	
C10	0.0411 (2)	0.7420 (2)	0.29918 (16)	0.0320 (4)	
H10	0.0039	0.8025	0.3516	0.038*	
C11	−0.0275 (2)	0.8040 (2)	0.17491 (16)	0.0336 (4)	
H11	−0.1079	0.9060	0.1432	0.040*	
C12	0.0253 (2)	0.7123 (2)	0.09853 (16)	0.0321 (4)	
C13	0.1472 (2)	0.5606 (2)	0.14326 (17)	0.0340 (4)	
H13	0.1808	0.5003	0.0905	0.041*	
C14	0.2181 (2)	0.50046 (19)	0.26736 (16)	0.0295 (3)	
H14	0.3016	0.3994	0.2978	0.035*	
C15	0.4973 (3)	0.03952 (19)	0.67375 (17)	0.0342 (4)	
H15A	0.3806	0.0217	0.6680	0.051*	
H15B	0.5895	−0.0505	0.7299	0.051*	
H15C	0.5430	0.0616	0.5926	0.051*	
C16	−0.0290 (3)	0.8027 (2)	0.63063 (19)	0.0402 (4)	
H16A	−0.0092	0.7243	0.7099	0.060*	
H16B	−0.1193	0.7945	0.5767	0.060*	
H16C	−0.0764	0.9021	0.6424	0.060*	

### Atomic displacement parameters (Å^2^)

**Table:**

	*U*^11^	*U*^22^	*U*^33^	*U*^12^	*U*^13^	*U*^23^
Cl1	0.0418 (2)	0.0380 (2)	0.0257 (2)	−0.01599 (18)	−0.00416 (16)	−0.00618 (17)
Cl2	0.0449 (3)	0.0568 (3)	0.0281 (2)	−0.0095 (2)	−0.00846 (19)	−0.0063 (2)
S	0.0416 (2)	0.0211 (2)	0.0340 (2)	−0.01348 (17)	0.00087 (18)	−0.00631 (16)
O2	0.0564 (9)	0.0358 (7)	0.0589 (9)	−0.0222 (6)	−0.0092 (7)	−0.0175 (7)
O1	0.0296 (6)	0.0221 (5)	0.0243 (5)	−0.0094 (4)	−0.0009 (4)	−0.0053 (4)
C1	0.0278 (7)	0.0208 (7)	0.0280 (8)	−0.0102 (6)	0.0006 (6)	−0.0052 (6)
C2	0.0253 (7)	0.0227 (7)	0.0289 (8)	−0.0111 (6)	0.0017 (6)	−0.0070 (6)
C3	0.0320 (8)	0.0257 (8)	0.0276 (8)	−0.0131 (6)	−0.0001 (6)	−0.0082 (6)
C4	0.0273 (8)	0.0315 (8)	0.0248 (8)	−0.0137 (6)	0.0000 (6)	−0.0059 (6)
C5	0.0257 (7)	0.0241 (8)	0.0296 (8)	−0.0088 (6)	0.0000 (6)	−0.0028 (6)
C6	0.0244 (7)	0.0225 (7)	0.0303 (8)	−0.0095 (6)	0.0019 (6)	−0.0061 (6)
C7	0.0241 (7)	0.0250 (7)	0.0245 (7)	−0.0112 (6)	0.0009 (6)	−0.0069 (6)
C8	0.0240 (7)	0.0215 (7)	0.0289 (8)	−0.0091 (6)	0.0012 (6)	−0.0052 (6)
C9	0.0247 (7)	0.0277 (8)	0.0262 (8)	−0.0125 (6)	0.0020 (6)	−0.0057 (6)
C10	0.0319 (8)	0.0309 (8)	0.0301 (8)	−0.0074 (7)	0.0007 (7)	−0.0094 (7)
C11	0.0298 (8)	0.0307 (8)	0.0325 (9)	−0.0066 (7)	−0.0017 (7)	−0.0031 (7)
C12	0.0272 (8)	0.0420 (10)	0.0241 (8)	−0.0136 (7)	−0.0016 (6)	−0.0041 (7)
C13	0.0346 (9)	0.0372 (9)	0.0308 (9)	−0.0114 (7)	0.0008 (7)	−0.0128 (7)
C14	0.0299 (8)	0.0272 (8)	0.0303 (8)	−0.0101 (6)	−0.0003 (6)	−0.0071 (7)
C15	0.0419 (9)	0.0230 (8)	0.0351 (9)	−0.0088 (7)	0.0016 (7)	−0.0082 (7)
C16	0.0454 (10)	0.0299 (9)	0.0426 (10)	−0.0085 (8)	0.0067 (8)	−0.0131 (8)

### Geometric parameters (Å, °)

**Table:**

Cl1—C4	1.7468 (17)	C8—C9	1.457 (2)
Cl2—C12	1.7358 (17)	C9—C10	1.399 (2)
S—O2	1.4862 (14)	C9—C14	1.402 (2)
S—C1	1.7672 (16)	C10—C11	1.380 (2)
S—C16	1.797 (2)	C10—H10	0.9300
O1—C7	1.3782 (19)	C11—C12	1.380 (3)
O1—C8	1.3794 (18)	C11—H11	0.9300
C1—C8	1.371 (2)	C12—C13	1.387 (3)
C1—C2	1.444 (2)	C13—C14	1.383 (2)
C2—C7	1.395 (2)	C13—H13	0.9300
C2—C3	1.401 (2)	C14—H14	0.9300
C3—C4	1.380 (2)	C15—H15A	0.9600
C3—H3	0.9300	C15—H15B	0.9600
C4—C5	1.403 (2)	C15—H15C	0.9600
C5—C6	1.389 (2)	C16—H16A	0.9600
C5—H5	0.9300	C16—H16B	0.9600
C6—C7	1.389 (2)	C16—H16C	0.9600
C6—C15	1.504 (2)		
			
O2—S—C1	107.20 (8)	C10—C9—C8	121.71 (15)
O2—S—C16	106.67 (10)	C14—C9—C8	119.94 (14)
C1—S—C16	97.03 (8)	C11—C10—C9	121.30 (16)
C7—O1—C8	106.66 (12)	C11—C10—H10	119.3
C8—C1—C2	107.57 (14)	C9—C10—H10	119.3
C8—C1—S	126.90 (12)	C10—C11—C12	118.87 (16)
C2—C1—S	125.25 (12)	C10—C11—H11	120.6
C7—C2—C3	119.54 (14)	C12—C11—H11	120.6
C7—C2—C1	104.68 (14)	C11—C12—C13	121.64 (16)
C3—C2—C1	135.77 (14)	C11—C12—Cl2	119.29 (14)
C4—C3—C2	116.18 (14)	C13—C12—Cl2	119.07 (14)
C4—C3—H3	121.9	C14—C13—C12	119.06 (16)
C2—C3—H3	121.9	C14—C13—H13	120.5
C3—C4—C5	123.34 (15)	C12—C13—H13	120.5
C3—C4—Cl1	118.85 (13)	C13—C14—C9	120.76 (16)
C5—C4—Cl1	117.80 (12)	C13—C14—H14	119.6
C6—C5—C4	121.32 (15)	C9—C14—H14	119.6
C6—C5—H5	119.3	C6—C15—H15A	109.5
C4—C5—H5	119.3	C6—C15—H15B	109.5
C7—C6—C5	114.60 (14)	H15A—C15—H15B	109.5
C7—C6—C15	121.71 (15)	C6—C15—H15C	109.5
C5—C6—C15	123.68 (14)	H15A—C15—H15C	109.5
O1—C7—C6	124.10 (14)	H15B—C15—H15C	109.5
O1—C7—C2	110.89 (13)	S—C16—H16A	109.5
C6—C7—C2	125.01 (15)	S—C16—H16B	109.5
C1—C8—O1	110.18 (14)	H16A—C16—H16B	109.5
C1—C8—C9	134.67 (15)	S—C16—H16C	109.5
O1—C8—C9	115.15 (13)	H16A—C16—H16C	109.5
C10—C9—C14	118.35 (15)	H16B—C16—H16C	109.5
			
O2—S—C1—C8	−144.91 (15)	C1—C2—C7—O1	−1.46 (17)
C16—S—C1—C8	105.17 (16)	C3—C2—C7—C6	−1.0 (2)
O2—S—C1—C2	28.29 (16)	C1—C2—C7—C6	178.09 (15)
C16—S—C1—C2	−81.64 (15)	C2—C1—C8—O1	0.14 (17)
C8—C1—C2—C7	0.79 (17)	S—C1—C8—O1	174.32 (11)
S—C1—C2—C7	−173.51 (12)	C2—C1—C8—C9	178.83 (16)
C8—C1—C2—C3	179.69 (17)	S—C1—C8—C9	−7.0 (3)
S—C1—C2—C3	5.4 (3)	C7—O1—C8—C1	−1.03 (16)
C7—C2—C3—C4	0.8 (2)	C7—O1—C8—C9	180.00 (12)
C1—C2—C3—C4	−177.97 (17)	C1—C8—C9—C10	−16.6 (3)
C2—C3—C4—C5	0.0 (2)	O1—C8—C9—C10	162.01 (14)
C2—C3—C4—Cl1	178.74 (12)	C1—C8—C9—C14	163.90 (17)
C3—C4—C5—C6	−0.7 (2)	O1—C8—C9—C14	−17.5 (2)
Cl1—C4—C5—C6	−179.45 (12)	C14—C9—C10—C11	−0.7 (2)
C4—C5—C6—C7	0.5 (2)	C8—C9—C10—C11	179.83 (15)
C4—C5—C6—C15	−178.23 (15)	C9—C10—C11—C12	1.4 (3)
C8—O1—C7—C6	−177.99 (14)	C10—C11—C12—C13	−1.0 (3)
C8—O1—C7—C2	1.57 (16)	C10—C11—C12—Cl2	179.16 (13)
C5—C6—C7—O1	179.82 (13)	C11—C12—C13—C14	−0.3 (3)
C15—C6—C7—O1	−1.4 (2)	Cl2—C12—C13—C14	179.60 (13)
C5—C6—C7—C2	0.3 (2)	C12—C13—C14—C9	1.1 (3)
C15—C6—C7—C2	179.10 (15)	C10—C9—C14—C13	−0.6 (2)
C3—C2—C7—O1	179.43 (13)	C8—C9—C14—C13	178.90 (15)

### Hydrogen-bond geometry (Å, °)

**Table:**

*D*—H···*A*	*D*—H	H···*A*	*D*···*A*	*D*—H···*A*
C15—H15A···O2^i^	0.96	2.52	3.216 (2)	130

Symmetry codes: (i) *x*, *y*−1, *z*.
